# Single-Shot Multicontrast X-ray Imaging for In Situ Visualization of Chemical Reaction Products

**DOI:** 10.3390/jimaging7110221

**Published:** 2021-10-23

**Authors:** Margarita Zakharova, Andrey Mikhaylov, Vitor Vlnieska, Danays Kunka

**Affiliations:** Institute of Microstructure Technology (IMT), Karlsruhe Institute of Technology (KIT), Hermann-von-Helmholtz-Platz 1, 76344 Eggenstein-Leopoldshafen, Germany; andrey.mikhaylov@kit.edu (A.M.); vitor.vlnieska@empa.ch (V.V.)

**Keywords:** X-ray imaging, Hartmann mask, phase-contrast imaging, chemical reaction, in situ

## Abstract

We present the application of single-shot multicontrast X-ray imaging with an inverted Hartmann mask to the time-resolved in situ visualization of chemical reaction products. The real-time monitoring of an illustrative chemical reaction indicated the formation of the precipitate by the absorption, differential phase, and scattering contrast images obtained from a single projection. Through these contrast channels, the formation of the precipitate along the mixing line of the reagents, the border between the solid and the solution, and the presence of the scattering structures of 100–200 nm sizes were observed. The measurements were performed in a flexible and robust setup, which can be tailored to various imaging applications at different time scales.

## 1. Introduction

Single-shot X-ray multicontrast imaging is a helpful tool for the visualization of inner structure evolution. Compared to conventional X-ray radiography, it offers additional contrast modalities such as phase and scattering (so-called dark-field). The phase-contrast image illustrates the wavefront irregularities caused by local differences in the real part of the refractive index [1]. Scattering contrast is obtained as a decrease in visibility of the wavefront modulation. It is attributed to the small-angle scattering caused by the structures well below the resolution of the imaging system [2].

Single-shot X-ray imaging methods based on a single optical element have the advantage of a simple and robust setup and do not require scanning the sample, in contrast to, for example, grating-based interferometry [2]. Various optical elements can be utilized, such as a two-dimensional absorption mask [3], microlens array [4,5,6,7], speckle generating diffuser [8], etc.

Although the single-shot implementation usually comes at the cost of reduced spatial resolution, such imaging modalities have several advantages over widely used grating-based interferometric X-ray imaging [2,9,10]. Relaxed requirements on the optical element precision and alignment eliminate the need for high aspect ratio gratings and increase the mechanical stability of the setup and the simplicity of its operation. Moreover, the setup with a single optical element manufactured on the low-absorbing substrate provides higher flux efficiency. Lower absorption in the optical element increases the photon statistics, which is crucial for time-resolved imaging, especially using monochromatic X-ray beams with a limited flux density. Such modalities are straightforward to be utilized for the retrieval of several contrast modalities from a single projection. Thus, it enables multimodal monitoring dynamic systems at different timescales: from real-time imaging [11] to fast imaging at the scale of microseconds [5,12,13,14].

Monitoring the dynamic systems in situ is a challenging but essential task aimed to enrich the understanding of the underlying mechanism and to define the timescale of the processes involved. Authors in several works have shown the possibility of performing in situ experiments using phase-contrast imaging (PCI) techniques, such as in-line holography PCI [15], propagation-based PCI [16], ptychography [17], etc.

Inverted Hartmann masks (iHMs) are simple but effective optical components that provide the wavefront modulation required for the subsequent retrieval of phase and scattering contrast modalities. They are represented by the arrays of periodic absorptive structures manufactured on a transmitting substrate. The iHMs provide high visibility when manufactured on low-absorbing materials and are easily scaled to a large field of view (FoV). They provide an increased photon flux at the detector due to the smaller area of the absorbing structures compared to a conventional mesh-like design. Inverted Hartmann masks manufactured on silicon substrates have already been used for the imaging of dynamic processes with intense white beams [3]. However, for monochromatic beams or laboratory X-ray setups, such masks cannot provide a high signal-to-noise ratio (SNR) due to the substantial absorption in the optical element. The manufacturing of the optical components on low-absorbing material such as graphite has been shown to improve visibility and flux efficiency [18]. The inverted Hartmann masks on graphite substrates have been successfully applied to phase-contrast imaging with an X-ray tube [19].

In this contribution, we present the application of the inverted Hartmann mask made on a low-absorbing graphite substrate to the time-resolved in situ visualization of chemical reaction products in a single-shot imaging setup. We performed real-time monitoring of copper hydroxide precipitation via the absorption, differential phase, and scattering contrast images obtained from a single projection. Small unresolvable changes in the refractive index were also detected by evaluating the scattering signal.

## 2. Materials and Methods

### 2.1. Chemical Reaction

**Preparation of the reagents.** Copper sulfate pentahydrate (99%) crystallized and sodium hydroxide in pellets (98%) were acquired from Sigma-Aldrich (Darmstadt, Germany). Chemicals were used as received. Copper sulfate pentahydrate solution was prepared as follows. In an Erlenmeyer, 10 mL of deionized water was added, followed by the addition of 2.497 g of copper sulfate pentahydrate (0.35 mol.L^−1^). The solution was stirred at room temperature until complete dissociation of the copper sulfate pentahydrate. Similarly, the sodium hydroxide solution was obtained by the addition of 0.400 g of sodium hydroxide (1.1 mol/L) to 10 mL of deionized water.

**Precipitation of copper hydroxide**. In an Eppendorf tube, 1 mL of Copper sulfate pentahydrate solution was added. Afterward, the Eppendorf was placed in the beam. The sodium hydroxide solution was connected to a peristaltic pump using a hose with an inner diameter of 190 µm. The other extremity of the hose was connected to the Eppendorf. The chemical reaction was achieved by pumping the aqueous solution of sodium hydroxide into the tube with copper(II) sulfate pentahydrate solution with a flow rate of 10 µL/sec. The products of the reaction were the aqueous solution of sodium sulfate and the copper(II) hydroxide as a precipitate:

(1) $$CuSO_{4}*5H_{2}O+2NaOH\to Na_{2}SO_{4}+Cu{\left(OH\right)}_{2}↓+5H_{2}O$$

During the precipitation of copper hydroxide, X-ray projection were acquired to perform in situ X-ray imaging of the chemical reaction.

### 2.2. X-ray Imaging

X-ray imaging was performed at the IPS imaging cluster of the KIT synchrotron facility. Quasimonochromatic X-rays with an energy of 17 keV and the energy bandwidth of 2% were incident on the inverted Hartmann mask of 50 µm period and the duty cycle of 0.5; the area of the mask was 5 × 5 cm. The mask consisted of an array of square gold pillars with a height of 30 µm. The mask was placed 112 cm away from the detector. The X-rays were detected by an Andor Neo 5.5 camera imaging an X-ray scintillator (LuAG) by lens coupling (magnification of 2.73). The effective pixel size was 2.4 µm. Exposure time per frame was 0.5 s with a frame rate of 1.5 Hz. The Eppendorf tube with copper sulfate pentahydrate solution was placed at 7.5 cm from the scintillator and connected to the pumping system located outside the FoV.

**Figure 1:**
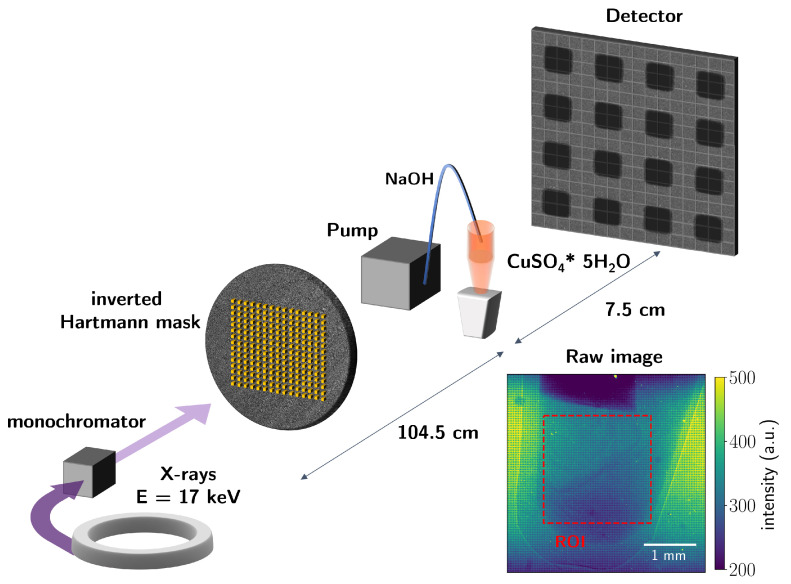
Experimental setup for in situ imaging of chemical reaction products by single-shot multicontrast X-ray imaging with the inverted Hartmann mask. After passing through the monochromator with the spectral bandwidth of 2%, the X-ray radiation had the central energy of 17 keV. The quasimonochromatic X-rays were incident on the inverted Hartmann mask. A tube with the copper sulfate pentahydrate solution was placed behind the mask. The sodium hydroxide solution was injected into the tube using the peristaltic pump connected to the tube with a hose.

The schematic representation of the setup is illustrated in Figure 1. The X-rays were passing through the inverted Hartmann mask, which introduced a periodic modulation to the wavefront. The periodically modulated wavefront was incident on the Eppendorf tube containing copper sulfate pentahydrate solution with NaOH being pumped into it at µL.sec^−1^ flow rate.

The retrieval of different contrast modalities was performed using SHWaveRecon software [20]. The software allows for phase retrieval using 2D multi-Gaussian fitting approach [4,21] or Fourier analysis [1]. The Fourier analysis approach was used in this work as it is more robust with noisy data [5]. The differential phase signals in two orthogonal directions were used to reconstruct the phase maps using the modified Southwell algorithm with 10 iterations [22].

## 3. Results

**Figure 2:**
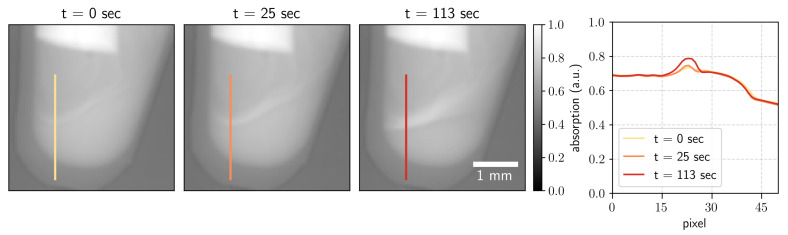
Absorption images for the measurement time *t* = 0, 25, and 113 s with the corresponding profiles along the indicated lines.

**Absorption contrast.** From each snapshot of the overlaying object-mask projection, three contrast modalities were retrieved. The absorption image was obtained as the ratio of the magnitudes of the zero-order (central harmonic) images with and without the sample [1,23]. The images obtained at measurement times 0, 25, and 113 s are shown in Figure 2. The line profiles in the images are on the right side of Figure 2. One can see how the absorption signal increases as the solid precipitate forms.

**Figure 3:**
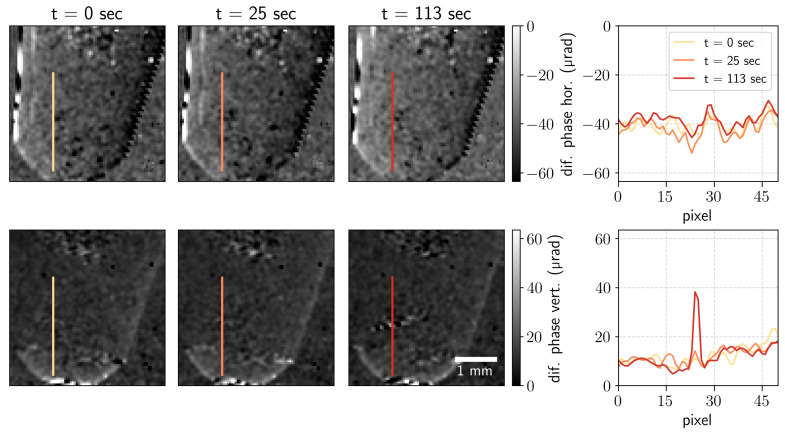
Differential phase images in two orthogonal directions for the measurement time *t* = 0, 25, and 113 s and the corresponding differential phase profiles along the indicated lines.

**Differential phase contrast.** The differential phase contrast (DPC) signal was retrieved by analyzing the shifts of the beam pattern in horizontal and vertical directions via the angle of the complex back-transformed first-order harmonics [5]. The DPC images show how the border from the solid precipitate forms. The profiles along the lines indicated in the images are plotted in Figure 3. One can see how the differential phase contrast grows with time in the indicated part of the Eppendorf tube. The formation of the solid precipitate changes the refractive index of the media, increasing the phase shift in the vertical direction. The phase-detection limit was 4 µrad. The angular resolution of the imaging setup can be improved by increasing the sample-to-detector distance.

**Figure 4:**
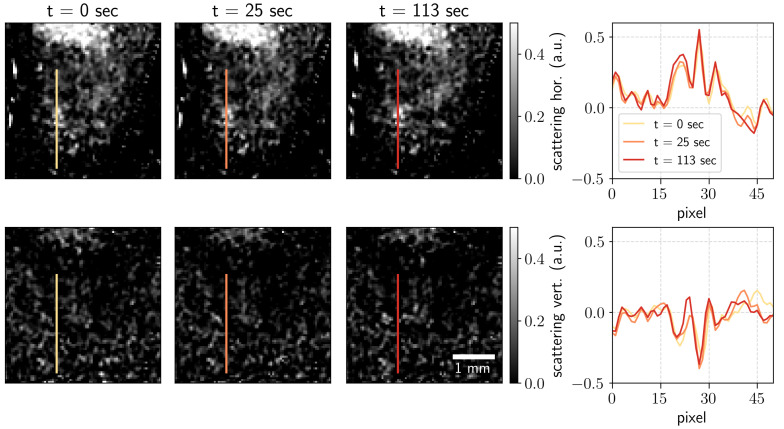
Scattering contrast images in two orthogonal directions for the measurement time *t* = 0, 25, and 113 s and the corresponding profiles along the indicated lines.

**Scattering contrast.** The scattering images were obtained from the decrease in intensity of the first-order harmonic normalized by the central harmonic in the Fourier domain [23]. The scattering contrast shows the signal from the unresolved features to be much smaller than the resolution of the imaging setup [2]. Scattering contrast images in two orthogonal directions are shown in Figure 4. The profiles along the lines indicated in the images are shown on the left. One can see that the profiles for scattering images do not change significantly with the measurement time. This might be because the small scatterers were already formed in the first seconds of the reactions that were not captured. Further reaction evolution with the precipitation of copper(II) hydroxide increases the number of pixels with a higher signal but does not significantly change the strength of the signal.

The unresolved absorption signal and the edge scattering can both contribute to the formation of the scattering contrast. The scattering signals obtained using Fourier analysis have been reported to exhibit the crosstalk correlation with the absorption signal due to the beam hardening for polychromatic sources [21] when the specimen exhibits high absorption [24]. This crosstalk can be neglected for the weakly absorbing microstructures; however, as the solid precipitate grows in volume, such signal pollution becomes more pronounced. For that reason, the linear decorrelation of the scattering signal and absorption was performed [5,25].

However, one can see that there is still a significant signal preserved in the scattering images for strongly absorbing structures such as the pumping hose. The curvatures of the hose refract at the angles both resolved and unresolved by the imaging setup. It can be seen from the direction scattering images that the tube is visible in the scattering in the horizontal direction, which is precisely the direction in which the curvature of the tube would refract. The ROI where the reaction occurred was not strongly affected. We did not observe a substantial scattering signal pollution in the vertical direction, the direction of the strongest refraction signal for the formed precipitate. If the object under study induces strong edge scattering, the pollution of the scattering contrast can be suppressed using special algorithms reported elsewhere [24].

The finite resolution of the imaging setup performs spatial ensemble average. In our case, the spatial resolution of the final image is defined by the mask period *P*. The dampening of the contrast averaged over the mask’s unit cell depends on the size of the scatterers presented in the sample [26]. The size sensitivity of the scattering contrast is defined by the setup organization [26]. The structure size probed in the setup is represented by the autocorrelation length ξ=λL/P, where λ is the wavelength, *P* the mask period, and *L* is the distance from the object to the detector. For the setup (Figure 1) used in this experiment, the correlation length was 110 nm.

**Figure 5:**
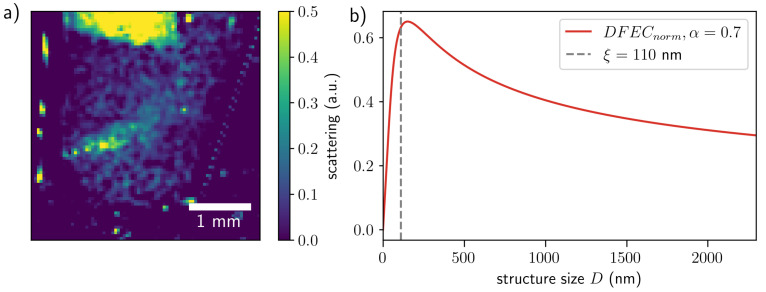
(**a**) Scattering contrast image at t=320 s (sum over the two orthogonal directions); (**b**) the dark-field extinction coefficient (DFEC) calculated using Equation (2) for the autocorrelation function according to the simplest general model (Equation (3)) versus scattering structure size. The roughness exponent α was set to 0.7 as obtained for densely packed structures [30].

The scattering intensity can be interpreted in terms of the dark-field extinction coefficient (DFEC) [26], which defines the scattering intensity and is related to the autocorrelation function G(ξ;D,α) as [27] follows:

(2) $$DFEC=\frac{3\pi^{2}}{\lambda^{2}}\varphi {\left|\Delta \chi \right|}^{2}\left[1-G(\xi ;D,\alpha )\right]\frac{D}{\xi },$$

where φ is the volume fractions of the scattering structure, Δχ the difference of complex refractive indices between the scattering material and the surrounding media, and *D* is the scattering structure size. The simplest form for the autocorrelation function corresponds to the model [28,29], where signal fluctuations are treated like surface roughness:

(3) $$G\left(\xi ;D,\alpha \right)=\exp\left[-{\left(\frac{\xi }{D}\right)}^{2\alpha }\right],$$

where α is the roughness exponent related to the fractal dimension of the scattering structure [29]. The roughness exponent α was calculated to be about 0.7 for densely packed structures [30]. We used the normalized DFEC $DFEC_{norm}=\left[1-G\left(\xi ;D,\alpha \right)\right]D/\xi$ to illustrate how the value of DFEC depends on the structure size (Figure 5b).

The scattering image obtained as a sum over the two orthogonal directions is shown in Figure 5a. The obtained values of the scattering signal are the average of the scattering intensity within the range of probed correlation lengths. As one can see from Figure 5b, the DFEC peaks close to the value of correlation length ξ=110 nm, thus demonstrating that the highest input to the signal is coming from the structures with sizes about 100–200 nm (location of the peak). The largest scatterer, which still can influence the value of the scattering signal, can be estimated as $\xi_{max}\approx \lambda L/PS\approx 2.3$ µm, where PS is the effective pixel size of the imaging setup.

## 4. Discussion

**Figure 6:**
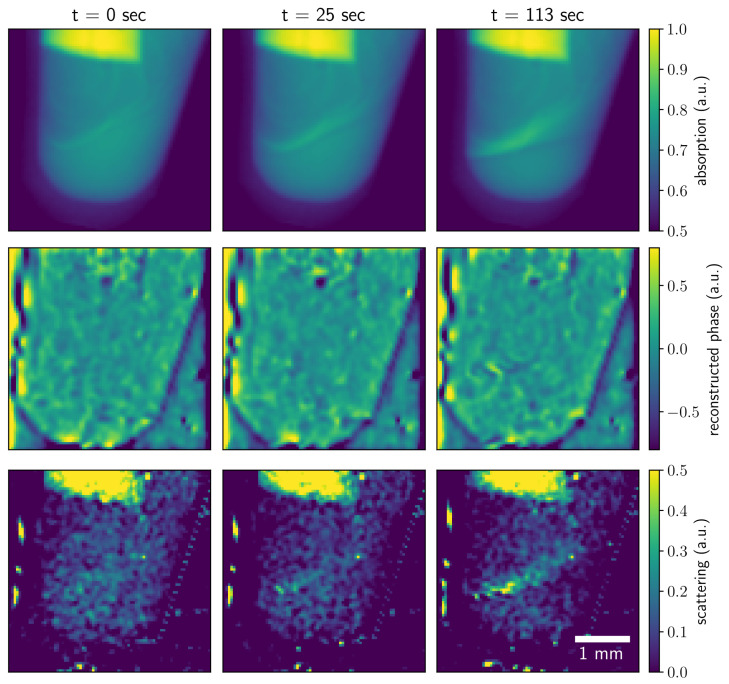
Absorption, reconstructed phase, and average scattering contrasts recorded at different measurement times (0, 25, and 113 s) illustrate the evolution of the chemical reaction.

The overview of the reaction is shown in Figure 6 and in the Supplemental Materials. Due to the technical restrictions of the setup, the pumping started several seconds before the image acquisition, such that a small amount of NaOH was already present in the tube. Thus, we performed the imaging of the course of the reaction but not its initiation.

The images for the three values of measurement time demonstrating the absorption, reconstructed phase, and the average scattering contrast are shown. One can see how each contrast modality illustrates the reaction evolution with time. As the aqueous sodium hydroxide solution is pumped into the copper(II) sulfate pentahydrate solution, the solid precipitate forms along the mixing line. The solid precipitate absorbs more X-rays than the aqueous solutions of reactants, which is reflected in the absorption contrast images. Copper(II) hydroxide has a different refractive index than the reagents. When a sufficient amount of the precipitate is formed, the difference in the introduced phase shift is detectable in the phase map. The average scattering contrast obtained from the scattering in two orthogonal directions illustrates the distribution of unresolved structures with sizes around 100–200 nm.

**Figure 7:**
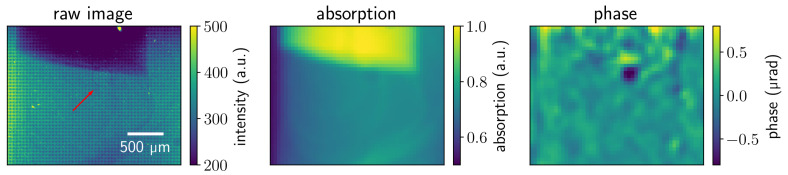
Raw, absorption, and reconstructed phase images in the area of an air bubble at *t* = 25 s. The arrow indicates the location of the bubble in the raw image.

The advantage of differential phase contrast for weakly absorbing structures can be clearly seen in Figure 7. It shows the cropped area of the air bubble pumped in the first second of the experiment. One can notice increased contrast on the bubble edges in the phase image in contrast to the raw and absorption images where it is barely visible.

**Figure 8:**
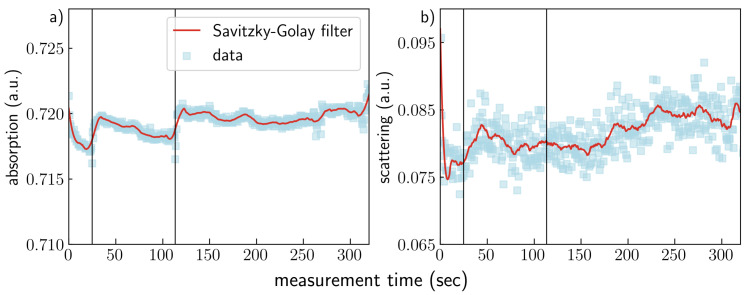
Evolution of the absorption (**a**) and scattering (**b**) contrast signals over the 320 s of measurement time. The data points represent the mean value of the signal in ROI indicated in Figure 1, and the red line is the result of data filtering with the Savitzky–Golay filter with a third-degree polynomial and a window of 21 data points. The gray and black vertical lines indicate the 25th and 113th seconds, respectively.

We illustrate the evolution of the absorption and scattering contrast channels over the 320 s in Figure 8. The data points represent the mean value of signal intensity in ROI indicated in Figure 1. The data were smoothed using the Savitzky–Golay filter with a third-degree polynomial and a window of 21 data points. The formation of the precipitate occurred during the first 320 s of the measurement as indicated by the jumps in the absorption signal (25 and 113 s of the measurement) (Figure 8a). Then, the signal stabilizes after the precipitate is formed. The evolution of the mean scattering contrast during the measurement is shown in Figure 8b. The signal starts to increase after the first precipitate formation at the 25th until the 50th second and then slightly decreases. The scattering contrast does not change significantly from 70 to 170 s and then starts to increase again. Note that there is no significant change in scattering signal, although the absorption signal shows the jump at 113 s. This implies that the volume fraction of scattering particles with sizes 100–200 nm keeps increasing, while the amount of resolved precipitate does not change significantly.

## 5. Conclusions

The single-shot multicontrast X-ray imaging setup used in this experiment allows for directly resolving the precipitates via absorption contrast and differential phase-contrast with the spatial resolution of 50 µm and the angular resolution of 4 µrad. Additionally, the scattering contrast enables tracking the submicron features. The formation of precipitate was completed after 130 s of the measurements, as seen from the stabilized absorption signal. The scattering signal continued to increase after forming a precipitate, indicating the increase in volume fraction of scattering structures with sizes of 100–200 nm. The short image acquisition time offered by single-shot X-ray imaging allows for monitoring of dynamic processes. Using brighter sources and detectors with a higher frame acquisition rate would further improve the time resolution.

The method is easy to implement and does not require a high degree of coherence. Flux-efficient inverted Hartmann masks are not susceptible to chromatic aberrations, enabling them to be used with polychromatic low-flux X-ray tubes. However, the adaptation to laboratory sources requires the special attention to factors such as the blur and magnification of the mask projection, which results in larger projected periods (lower spatial resolution). For such applications, the object-before-mask geometry of the setup can be advantageous. Moreover, the polychromaticity of the radiation can lead to the spurious signal in scattering images, which can be suppressed with the decorrelation algorithms [5,24].

Hartmann masks are easy to handle and do not require precise alignment, making the setup flexible and straightforward to tailor for different applications. The single-shot multicontrast X-ray imaging with the inverted Hartmann mask can be used to study dynamic processes at different time scales. Only the source flux density and the detector frame limit the time resolution. Such a robust and flexible approach can have various applications in materials science and medical imaging.

## Data Availability

The data presented in this article are available on request from the corresponding authors.

## Supplementary Materials

The following are available online at https://www.mdpi.com/article/10.3390/jimaging7110221/s1, Video S1: In situ visualization of chemical reaction products formation.

## Abbreviations

The following abbreviations are used in this manuscript:

FoV	Field of View
ROI	Region of Interest
iHM	inverted Hartmann mask
2D	two-dimensional
SNR	Signal-to-Noise Ratio
DPC	Differential Phase Contrast
